# In-vivo T1 cardiovascular magnetic resonance study of diffuse myocardial fibrosis in hypertrophic cardiomyopathy

**DOI:** 10.1186/1532-429X-16-28

**Published:** 2014-04-25

**Authors:** Wessel P Brouwer, Emma N Baars, Tjeerd Germans, Karin de Boer, Aernout M Beek, Jolanda van der Velden, Albert C van Rossum, Mark BM Hofman

**Affiliations:** 1 Department of Cardiology, ICaR-VU, VU University Medical Center, De Boelelaan 1117, Amsterdam 1081 HV, the Netherlands; 2 Department of Physiology, ICaR-VU, VU University Medical Center, Amsterdam, the Netherlands; 3 Interuniversity Cardiology Institute of the Netherlands, Utrecht, the Netherlands; 4 Department of Physics and Medical Technology, ICaR-VU, VU University Medical Center, Amsterdam, the Netherlands

**Keywords:** T1 mapping, CMR, Diffuse fibrosis, HCM, Extracellular volume fraction

## Abstract

**Background:**

In hypertrophic cardiomyopathy (HCM), autopsy studies revealed both increased focal and diffuse deposition of collagen fibers. Late gadolinium enhancement imaging (LGE) detects focal fibrosis, but is unable to depict interstitial fibrosis. We hypothesized that with T1 mapping, which is employed to determine the myocardial extracellular volume fraction (ECV), can detect diffuse interstitial fibrosis in HCM patients.

**Methods:**

T1 mapping with a modified Look-Locker Inversion Recovery (MOLLI) pulse sequence was used to calculate ECV in manifest HCM (n = 16) patients and in healthy controls (n = 14). ECV was determined in areas where focal fibrosis was excluded with LGE.

**Results:**

The total group of HCM patients showed no significant changes in mean ECV values with respect to controls (0.26 ± 0.03 vs 0.26 ± 0.02, p = 0.83). Besides, ECV in LGE positive HCM patients was comparable with LGE negative HCM patients (0.27 ± 0.03 vs 0.25 ± 0.03, p = 0.12).

**Conclusions:**

This study showed that HCM patients have a similar ECV (e.g. interstitial fibrosis) in myocardium without LGE as healthy controls. Therefore, the additional clinical value of T1 mapping in HCM seems limited, but future larger studies are needed to establish the clinical and prognostic potential of this new technique within HCM.

## Background

Hypertrophic cardiomyopathy (HCM) is a genetic heart disease, characterized by unexplained left ventricular (LV) hypertrophy, often accompanied by myocardial fibrosis [1]. Post-mortem studies revealed that fibrosis is either present as focal scarring, or diffusely by intercellular deposition of collagen fibers [2]. Histology revealed increased amounts of fibrous tissue not solely in the interventricular septum, but also in the LV free wall and even in the right ventricle [3]. However, pathology studies reported inconsistent quantities of interstitial fibrosis, most likely as a result of differences in staining techniques and the heterogeneous nature of HCM [3-5]. In-vivo, focal fibrosis can be assessed non-invasively with cardiovascular magnetic resonance late gadolinium enhancement imaging (CMR-LGE) [6]. This technique however is limited by its reliance on relative differences of gadolinium (Gd) uptake, assuming that areas with the lowest concentration of Gd consist of normal myocardium. T1 mapping on the other hand is a CMR technique that allows absolute T1 value measurements in any region of the myocardium, enabling the calculation of the myocardial extracellular volume fraction (ECV), which represents the amount of interstitial fibrosis [7,8]. We hypothesized that non-enhanced regions in manifest HCM contain interstitial fibrosis that remain undetected by current LGE imaging. Therefore, we applied T1 mapping before and after contrast administration to calculate ECV in these non-enhanced regions. ECV values were compared to those in healthy controls.

## Methods

### HCM patients

The study was approved by the institutional research ethics board and written informed consent was obtained from all study participants. Patients were included in the study when they showed a LV wall thickness (WT) ≥15 mm as assessed with CMR in the absence of conditions associated with increased loading of the heart [9]. HCM mutation analysis was performed in a subset of patients, according to standard sequencing schemes. Exclusion criteria were a history of coronary artery disease (CAD) and any contra-indication for CMR such as an implanted pacemaker or internal cardioverter defibrillator (ICD) or claustrophobia.

### Healthy controls

The subjects in the control group were either healthy volunteers or genotype negative family members of index HCM patients, without a history of CAD, valve disease or hypertension.

### CMR acquisition

CMR studies were performed on a 1.5 Tesla whole body MRI system (Magnetom, Avanto, Siemens, Erlangen, Germany). First, standard scouts were obtained, followed by long axis and short axis cine images using a retrospective gated, steady state free precession (SSFP) gradient echo sequence. In vivo T1 mapping was performed at a mid-ventricular short axis slice before, ~8 minutes and ~20 minutes after the infusion of a bolus (0.2 mmol/kg) of gadolinium-diethylene triamine pentaacetic acid (Gd-DTPA) (Magnevist, Scheringen, Berlin, Germany) or gadoterate meglumine (Gd-DOTA) (Dotarem, Guerbet, Roissy CdG, France). Both agents are paramagnetic contrast agents with a similar extracellular distribution and a similar R1 relaxivity [10]. A single breath-hold, modified Look-Locker Inversion Recovery (MOLLI) pulse sequence with ECG-gating was applied to determine absolute T1 values of the myocardium at all three time points. Eleven images with various inversion delays were obtained with a 3-3-5 scheme within 17 heart beats [11]. LGE-imaging with full LV coverage was performed approximately ten minutes after gadolinium infusion, using an inversion recovery spoiled gradient echo sequence with magnitude image reconstruction. Images were corrected for surface coil inhomogeneities by a PD-weighted 3D prescan as implemented in the standard software. The inversion time was set to null the CMR signal of normal appearing myocardial tissue, typically at 250 ms. Slice positions of mid-ventricular T1 mapping images and corresponding mid-ventricular LGE images were identical.

### Offline LV and LGE-analysis

Left ventricular mass was calculated by manual delineation of endocardial and epicardial borders at the end-diastolic phase on all short axis slices and was indexed for body surface area (BSA). LGE-images were visually scored on the presence of focal fibrosis. When no focal fibrosis was present, the entire myocardial short axis area of the corresponding T1 map was selected to determine the average myocardial T1 value.

When focal fibrosis was present (LGE-positive), areas of non-enhanced myocardial tissue were selected. A threshold algorithm was applied on the magnitude LGE images to select myocardium with a signal intensity (SI) <20% of maximal SI at the core area of focal fibrosis. Normally, a 50% threshold is applied to select enhanced myocardial tissue, a full width half maximum (FWHM) algorithm [12]. To ensure the selection of clear non-enhanced tissue, a <20% threshold was applied. In these regions of non-enhanced myocardium, a region of interest (ROI) was drawn on the corresponding T1 map to determine T1 values. When LGE was present on a different level than the mid-ventricular T1 mapping slice, signal intensities of these LGE-positive areas were used to set the threshold for the mid-ventricular LGE slice. Dedicated software was used for this analysis (Mass, Medis, Leiden, the Netherlands).

### T1 mapping analysis and ECV calculation

**Figure 1:**
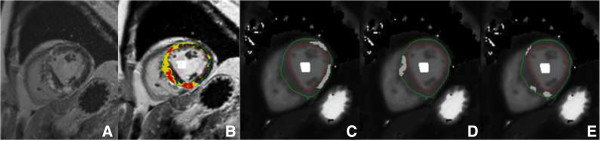
**The selection of myocardial regions with different signal intensities in a LGE-positive HCM patient.** Image **A** represents a short axis LGE image of a HCM patient, showing extensive focal fibrosis in the myocardium (white areas). The maximum signal (max SI) was determined by drawing a small ROI in the core of the region with focal fibrosis. Image **B**, which is the identical CMR image as (A) but with a segmentation overlay, shows non-enhanced myocardium (SI <20% max SI), intermediate enhanced myocardium in yellow (>20 and <50% of max SI) and enhanced myocardium in red (SI >50% of max SI) using the thresholding algorithm. Image **C**, **D**, and **E** show manually drawn ROI’s on corresponding T1 maps for non-enhanced, intermediate enhanced, and enhanced myocardium, respectively. LGE = late gadolinium enhancement. SI = signal intensity. ROI = region of interest.

Offline T1 maps were calculated from the MOLLI images [13] with manual motion correction when deemed necessary [14]. ROI’s were drawn in non-enhanced myocardium in LGE images and an additional ROI was placed at the center of the LV blood pool, as shown in Figure 1. These ROI’s were copied to corresponding T1 maps for all experiments. Relaxation rates (R1 = 1/T1) were calculated for myocardium and blood pool for both pre-contrast and post-contrast T1 mapping experiments. Subsequently, the myocardial partition-coefficient of the contrast agent was calculated by least-squares linear regression of myocardial R1 values versus blood pool R1 values over the 3 time points assuming a dynamic equilibrium of contrast concentration between blood and extracellular space [15]. Finally, ECV, reflecting the amount of interstitial fibrosis, was calculated using a correction for blood hematocrit [7,15]. In all subjects, hematocrit values were obtained immediately before or after the CMR acquisition.

To demonstrate that T1 mapping discriminates adequately areas of clear enhancement, intermediate enhancement and no enhancement, ECV was determined in LGE-positive HCM patients in areas with focal fibrosis (SI of LGE >50% of maximum, equal to the FWHM algorithm), within an area of intermediate SI (>20% and <50% of maximum SI) and within an area of non-enhanced myocardium (see Figure 1).

In subjects with no focal fibrosis, also a segmental analysis of the T1 maps and subsequent ECV was performed using 6 segments (inferoseptal, anteroseptal, anterior, anterolateral, inferolateral and inferior) according to the 17-segment model of the American Heart Association (AHA) [16].

### Statistical analysis

All statistical analyses were performed in SPSS version 15.0 (SPSS Inc., Chicago, IL, USA).

Data are expressed as mean ± standard deviation (SD). Continuous data were compared using a two-sided Student’s *t*-test or non-parametric tests when appropriate. For multiple comparisons, analysis of variance (ANOVA) with Bonferroni correction was used. Proportions were compared using a Chi-square test. Univariate regression analysis was performed to evaluate the relation of age, gender, and the presence of LGE with ECV. Factors that showed a significant association in this univariate analysis were subsequently evaluated in a multivariate linear regression analysis. Mutation status was not evaluated in the model, since not all HCM patients underwent prior genetic testing. P-values <0.05 were considered statistically significant.

## Results

### Patient characteristics

In total 20 HCM patients were included in the study, with a mean age of 50 ± 14 years and 80% male. As a result of a medical history of hypertension, 4 out of 20 HCM patients were excluded from the primary analysis. The remaining 16 HCM patients were predominantly male (n = 12) with mean age 47 ± 14 years. Fourteen healthy subjects served as controls (8 male, mean age 48 ± 15 years). Baseline characteristics of HCM patients and controls are outlined in Table 1. Both groups were comparable regarding age and gender. As shown, the majority of HCM patients had mutation positive status and received cardiac medication. Blood hematocrit (L/L) levels were significantly higher in HCM compared to controls (0.45 ± 0.04 vs 0.42 ± 0.02, p = 0.007).

**Table 1:** Baseline characteristics of HCM patients and controls

	**HCM (n = 16)**	**Controls (n = 14)**	**p-value**
Age (y)	47 ± 14	48 ± 15	0.82
Gender (m/f)	12/4	8/6	0.44
Mutation in sarcomeric gene	*- MYBPC3* (6, 38%)		
	*- MYH7* (1, 6%)		
	*- TNNT2* (2, 13%)		
	- Excluded (3, 19%)	- Excluded (11, 79%)	
	- Unknown (4, 25%)	- Unknown (3, 21%)	
Systolic BP (mmHg)	132 ± 12	132 ± 26	0.96
Diastolic BP (mmHg)	75 ± 11	77 ± 9	0.67
Heart rate (BPM)	63 ± 13	68 ± 10	0.21
BSA (kg/m^2^)	2.0 ± 0.17	1.9 ± 0.19	0.10
Medication	Beta-blocker (8, 50%)	None	
	Ca-antagonist (1, 6%)		
Hematocrit (L/L)	0.45 ± 0.04.	0.42 ± 0.02	0.007

*MYBPC3, MYH7* and *TNNT2* genes encoding the sarcomeric proteins myosin binding protein C, myosin heavy chain and troponin T, respectively. BP = blood pressure. BPM = beats per minute. BSA = body surface area.

### CMR findings

Mean indexed LV mass in HCM patients was 83 ± 37 g/m^2^ and mean maximal LV wall thickness was 18 ± 3 mm. Nine (56%) HCM patients showed areas of focal fibrosis, of which eight displayed enhancement at the mid-ventricular LGE image. The remaining patient showed regional contrast enhancement at LV regions that were non-corresponding with the mid-ventricular T1 map. None of the healthy controls showed signs of late LGE in the heart muscle.

### T1 mapping analysis

**Figure 2:**
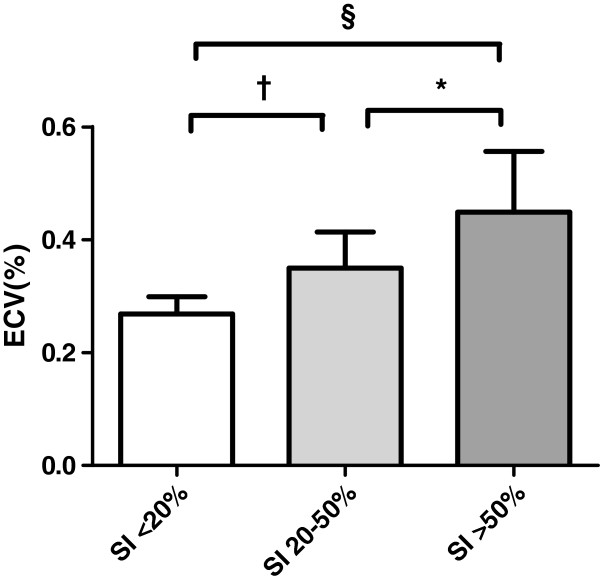
**ECV values in regions with different myocardial enhancement at LGE-images in HCM patients with focal fibrosis.** Regions in the core of focal fibrosis (SI >50%), apparent normal regions (SI <20%), and regions with intermediate enhancement (SI 20-50%) *p < 0.05, † p < 0.01, § p < 0.001. ECV = Extracellular volume fraction. SI = signal intensity.

Motion correction was applied in 9 T1 maps in 6 subjects (4 HCM, 2 controls) to correct for respiratory motion. Figure 2 shows the ECV values in LGE positive HCM patients in myocardial regions with SI <20%, SI 20-50% and SI >50%. Regions with SI >50% showed significantly higher ECV values with respect to regions with a SI between 20-50% and regions with a SI <20% (0.45 ± 0.11.vs 0.35. ± 0.06, p = 0.03 and 0.45 ± 0.11 vs 0.27 ± 0.03, p < 0.001, respectively). Furthermore, myocardium with SI 20-50% on LGE imaging showed a significantly higher ECV value than myocardium with a SI <20% (p = 0.003).

#### Difference in mean ECV values between groups

**Figure 3:**
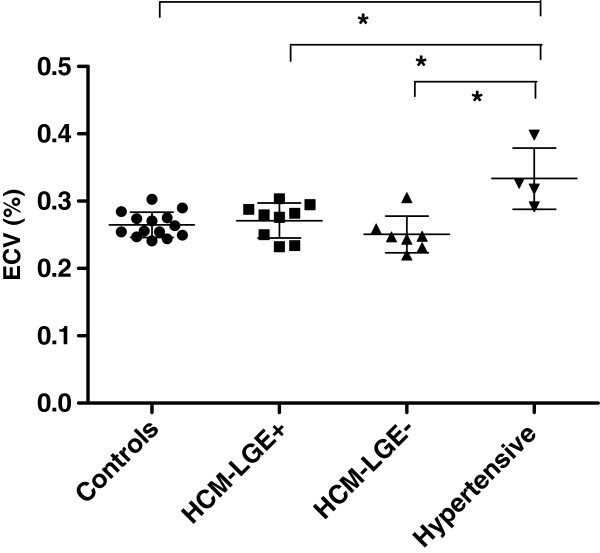
**Extracellular volume fraction (ECV) values in non-enhanced myocardium in HCM patients and controls.** The group of HCM patients is subdivided into patients with focal fibrosis on LGE (HCM-LGE+) and without focal fibrosis (HCM-LGE-). Notice that solely the group of ‘hypertensive HCM’ patients shows a significantly higher ECV compared to the other groups. ECV = extracellular volume fraction. HCM = hypertrophic cardiomyopathy. LGE = late gadolinium enhancement. * p < 0.05.

Analysis and comparison of ECV values in non-enhanced myocardium in HCM patients and controls resulted in the following; no differences were observed in ECV values between the total group of HCM patients and controls (0.26 ± 0.03 vs 0.26 ± 0.02, p = 0.83), between LGE positive HCM patients and controls (0.27 ± 0.03 vs 0.26 ± 0.02, p = 0.41), and between LGE negative HCM patients and controls (0.25 ± 0.03 vs 0.26 ± 0.02, p = 0.17). Besides, ECV differences between LGE positive and LGE negative HCM patients were non-significant (p = 0.12). Results are depicted in Figure 3.

#### Segmental analysis of ECV

Within HCM patients without LGE, ANOVA analysis with Bonferroni correction revealed no significant differences in ECV between segments (p = 0.63). In controls however, ECV values differed significantly (p = 0.01), with both inferoseptal and anteroseptal segments containing highest ECV values (both 0.28 ± 0.02) and anterior and anterolateral lowest values (both 0.25 ± 0.02). When we compared corresponding segments of LGE negative HCM patients and controls, there were no significant differences in ECV values (see Table 2). When segments containing ROI’s with SI <20% in LGE positive HCM patients were included in the segmental analysis, results remained non-significant (data not shown).

**Table 2:** Segmental values of myocardial extracellular volume fraction in HCM LGE negative patients and controls

**ECV per segment**	**HCM-LGE- (n = 7)**	**Controls (n = 14)**	**p-value**
Inferoseptal	0.26 ± 0.02	0.28 ± 0.02	0.08
Anteroseptal	0.26 ± 0.03	0.28 ± 0.02	0.09
Anterior	0.25 ± 0.03	0.25 ± 0.02	0.68
Anterolateral	0.24 ± 0.03	0.25 ± 0.02	0.20
Inferolateral	0.24 ± 0.04	0.26 ± 0.02	0.44
Inferior	0.24 ± 0.04	0.27 ± 0.03	0.13

#### Hypertrophic and non-hypertrophic segments of the left ventricle

When ECV values between non-enhanced hypertrophic (LVWT ≥15 mm, n = 8) and non-hypertrophic (LVWT <15 mm, n = 56) segments were compared in the group of HCM patients, hypertrophic segments showed significantly higher ECV values (0.29 ± 0.03 vs 0.26 ± 0.04, p = 0.02). Of the 8 hypertrophic segments, 3 were located at the inferoseptum, 3 at the anteroseptum and 2 anterior.

### Factors associated with mean ECV

For the total study population (HCM patients and controls combined, n = 30), age, gender and the presence of LGE were not significantly associated with ECV in univariate regression analysis (R = 0.22, p = 0.24, R = 0.12, p = 0.53 and R = 0.25, p = 0.19, respectively). Interestingly, when we added the excluded 4 HCM patients to the analysis, hypertension was independently and strongly associated with ECV (R = 0.66, p < 0.001). Off note, two of these four patients carried a sarcomeric mutation, one had extreme LVWT (24 mm) and one patient showed patchy LGE and had a LVWT of 21 mm. Within the group of HCM patients only, there were no significant relationships between LV wall thickness and LV mass with ECV (R = 0.33, p = 0.22 and R = 0.36, p = 0.17). Also, there was no difference in ECV between genotype positive HCM patients and HCM patients in whom a sarcomeric mutation was excluded by genetic analysis (0.26 ± 0.03 vs 0.24 ± 0.02, p = 0.31).

## Discussion

The major outcome of this CMR study using T1 mapping was that in hypertrophic cardiomyopathy areas without late gadolinium enhancement showed a similar extracellular volume fraction (ECV) compared to controls. This is in line with a study by Ugander et al. [17], who reported non-elevated ECV in ‘normal appearing’ myocardium in non-ischemic cardiomyopathies. Study results are not directly comparable however, since the patient population in present study was defined more precisely (HCM only) and thresholding was used to select ROI’s in LGE positive HCM patients instead of visual interpretation.

The second observation in this study was that besides comparable global (mean) ECV values, also segmental ECV values were virtually similar between HCM patients and controls (see Table 2). Interestingly, in agreement with the general observation that focal fibrosis typically occurs in areas with LV hypertrophy, we observed highest ECV values in hypertrophic LV segments. It must be noted however that the majority of these segments were located at the septum, which also showed highest ECV values in healthy control subjects (Table 2). The ECV value observed in our control group was similar to values reported in earlier studies using a similar MOLLI-sequence for T1 mapping before and after contrast administration, and the standard deviation was rather small [18,19].

To demonstrate that absolute T1 measurements reliably discriminated areas with different signal intensities on qualitative LGE images in our study, myocardium was stratified into low, intermediate and high enhancement on LGE images. Figure 2 demonstrated that the translation between these quantitative (T1 mapping) and qualitative (LGE) imaging techniques was valid in differentiating myocardial tissue with various collagen concentrations.

In this study, we applied ECV assessment after a bolus of gadolinium, in contrast to Flett et al. [7], who used continuous contrast infusion to obtain a steady state concentration of contrast agent between myocardium and blood. Jerosch-Herold et al. [15] showed that a dynamic equilibrium of contrast between blood and extracellular space is reached after a minimum delay of four minutes following bolus infusion. Such a dynamic equilibrium also allows ECV assessment, as confirmed by the study of Chow et al. [20] and White et al. [21]. In this study, a time delay of about 8 minutes was applied before the first post-contrast T1 experiment. A clear indication for the presence of a dynamic equilibrium was the observed linear relation of R1 values between myocardium and blood over all three time points (data not shown), without obvious deviation.

In our study, the factors age, gender, and the presence of LGE showed no significant association with the extracellular volume fraction in non-enhanced myocardium. The observation that age was non-significant related to ECV is in line with the study by Ugander et al. [17], in which only a modest correlation (r = 0.28) was observed in a larger patient group. We observed no gender-specific differences in ECV, either on a global or on a segmental level, in contrast to Sado et al. [18], who found higher ECV in the septum of women. Although hypertension is a strict exclusion criterion for the diagnosis HCM [1], we performed a secondary analysis with four ‘hypertensive HCM’ patients, of which two had genotypic diagnosis of HCM and two had a phenotype strongly suggesting HCM (extreme LVWT and/or ‘patchy’ fibrosis) [1,22]. Hypertension appeared a strong and independent predictor of ECV, which is in agreement with a recent study by Coelho-Filho et al. [23]. They reported also on an elevated ECV in hypertensive patients compared to healthy controls. Apparently, increased systemic pressure importantly contributes to diffuse fibrosis formation of the heart, possibly irrespective of the underlying condition of the cardiac muscle involved. Future studies are needed however to address this issue.

As stated before, the group of HCM patients showed no differences in ECV in non-enhanced myocardium with respect to controls. This contradicts studies that reported increased myocardial collagen synthesis in HCM [24], which was even seen in a pre-hypertrophic state [25]. Our study results also disagree with various reports of increased deposition of diffuse fibrosis throughout the entire LV in post-mortem HCM hearts [4,5]. These discrepancies may be explained by the following; First, T1 mapping may lack the sensitivity to detect subtle interstitial changes, which can be visualized with microscopy or indirectly demonstrated by molecular analysis. Secondly, the fibrotic burden of the myocardium in our mainly asymptomatic HCM patients is likely to be less compared to the explanted hearts of ‘end-stage patients [2-4].

### Limitations

Since this study was conducted in a relatively low number of HCM patients and controls, results should be interpreted with care. Nevertheless, mean ECV in healthy subjects was comparable with control values previously reported by other study groups [18,19] and standard deviations were relatively small, indicating reliable methodology. The sensitivity of T1 mapping for motion is known to generate some blurring of images and therefore decreased reliability of measurements, since through-plane motion was not corrected for. To minimize these effects of through-plane motion, we assessed ECV values in regions without enhancement with a maximum size. Another limitation of the study is the use of a single mid ventricular short axis slice for ECV assessment. Whole heart mapping would have been preferable since HCM is thought to display (focal) fibrosis throughout the entire LV. Moreover, two types of contrast agents were applied in this prospectively designed study. The subset of control patients in whom sarcomeric mutations were excluded received Gd-DTPA, since they also served as controls in another study. In the remainder of controls and patients, the newer Gd-DOTA agent was used. The impact on ECV calculation is considered non-significant however, since the relevant pharmokinetics of both contrast agents are similar, as well as their relaxivity [10]. The ECV determination is insensitive to small changes in absolute relaxivity of the agent, as both blood and tissue T1 values are changed. Finally, a phase sensitive inversion recovery (PSIR) reconstruction has been shown superior to magnitude reconstruction in defining areas in LGE images [26]. However, in our study optimal nulling and selection of ROI’s using a low threshold method minimized these subtle effects.

## Conclusions

This CMR study showed that HCM patients have a similar extracellular volume fraction, in myocardium without LGE as healthy controls. The additional clinical value of T1 mapping in HCM seems therefore limited, but future larger studies are needed to establish the clinical and prognostic potential of this new technique within HCM.

## Abbreviations

CMR: Cardiovascular magnetic resonance; LGE: Late gadolinium enhancement; ECV: Myocardial extracellular volume fraction; MOLLI: Modified look-locker inversion recovery; HCM: Hypertrophic cardiomyopathy; LV: Left ventricular; PSIR: Phase sensitive inversion recovery; BSA: Body surface area; FWHM: Full width at half maximum; SI: Signal intensity; ROI: Region of interest; CVF: Collagen volume fraction; SCD: Sudden cardiac death; MYBPC3: Cardiac myosin-binding protein C; TNNT2: Cardiac muscle troponin T; MYH7: Cardiac muscle β-myosin heavy chain; SSFP: Steady state free precession; WT: Wall thickness; CAD: Coronary artery disease.

## Competing interests

The authors declare that they have no competing interests.

## Authors’ contribution

WPB writing of the manuscript, subject recruitment, data analysis, critical appraisal. EB has been involved in data acquisition, data analysis and drafting of the manuscript. TG involvement in data analysis, drafting and critical appraisal of the manuscript. KB involved in patient recruitment, design of the study and critical appraisal of the manuscript. AMB participated in the design of the study, in optimizing scan sequences and critical appraisal of the manuscript. JV participated in rationale and design of the study, statistical analysis and drafting of the manuscript. ACR conceived of the study and helped to draft the manuscript and gave (together with MBM) final approval for publishing. MBMH participated in the design of the study, in scan optimization, data acquisition, data analysis, writing of the manuscript and final approval for publishing. All authors read and approved the final manuscript.
